# Maternal dyslipidemia and altered cholesterol metabolism in early pregnancy as a risk factor for small for gestational age neonates

**DOI:** 10.1038/s41598-021-00270-1

**Published:** 2021-10-26

**Authors:** So Yeon Kim, Seung Mi Lee, Go Eun Kwon, Byoung Jae Kim, Ja Nam Koo, Ig Hwan Oh, Sun Min Kim, Sue Shin, Won Kim, Sae Kyung Joo, Errol R. Norwitz, Young Mi Jung, Chan-Wook Park, Jong Kwan Jun, Man Ho Choi, Joong Shin Park

**Affiliations:** 1 Department of Obstetrics and Gynecology, Seoul National University College of Medicine, 101 Daehak-Ro, Jongno-Gu, Seoul, 03080 Korea; 2 Department of Obstetrics and Gynecology, University of Ulsan College of Medicine, Asan Medical Center, Seoul, Korea; 3 Molecular Recognition Research Center, Korea Institute of Science and Technology, 5 Hwarang-ro 14-gil, Seoul, 02792 Korea; 4 Department of Obstetrics and Gynecology, Seoul Metropolitan Government Seoul National University Boramae Medical Center, Seoul, Korea; 5 Seoul Women’s Hospital, Incheon, Korea; 6 Department of Laboratory Medicine, Seoul National University College of Medicine, Seoul, Korea; 7 Department of Laboratory Medicine, Seoul Metropolitan Government Seoul National University Boramae Medical Center, Seoul, Korea; 8 Department of Internal Medicine, Seoul National University College of Medicine, Seoul, Korea; 9 Department of Internal Medicine, Seoul Metropolitan Government Seoul National University Boramae Medical Center, Seoul, Korea; 10 Department of Obstetrics and Gynecology, Tufts University School of Medicine, Boston, MA USA

**Keywords:** Pregnancy outcome, Dyslipidaemias, Intrauterine growth

## Abstract

We evaluated the relationship between maternal cholesterol levels and its biologically active precursors and metabolites in the first trimester and subsequent risk for small-for-gestational-age birthweight (SGA). This is a secondary analysis of a prospective cohort study which enrolled healthy singleton pregnancies (n = 1337). Maternal fasting blood was taken in the first trimester and followed up till delivery. The lipid parameters were compared between women who delivered SGA neonates (SGA-group, birthweight < 10th percentile, n = 107) and women who did not (non-SGA-group, n = 1230). In addition, metabolic signatures of cholesterol were evaluated in a subset consisting of propensity-score matched SGA (n = 56) and control group (n = 56). Among lipid parameters, maternal high-density lipoprotein cholesterol (HDL-C) levels were significantly lower in SGA-group than in non-SGA-group (*p* = 0.022). The risk for SGA was negatively correlated with maternal serum HDL-C quartiles (*p* = 0.003), and this association remained significant after adjustment for confounding variables. In metabolic signatures of cholesterol, the cholesterol/lathosterol ratio in SGA-group was significantly higher than non-SGA-group [(2.7 (1.6–3.7) vs. 2.1 (1.5–2.9), respectively; *p* = 0.034)], suggesting increased endogenous cholesterol biosynthesis. We demonstrated that dyslipidemia and increased cholesterol biosynthesis led to delivery of SGA neonates even in early pregnancy.

## Introduction

Small-for-gestational-age birthweight (SGA) is defined as birth weight is less than 10 percentile^1^. Although a fraction of SGA neonates are constitutionally small and not related to adverse neonatal outcomes, SGA neonates are often designated as having fetal growth restriction (FGR)^1^. The definition of FGR implies pathologic condition which results in not fulfilling fetal growth potential and increase perinatal mortality and morbidities^2–4^. After the introduction of Baker's hypothesis, the importance of FGR has been emphasized, as FGR could be associated with an increased incidence of metabolic syndrome, such as obesity, cardiovascular disease, and diabetes in adulthood^5^.

SGA can be caused not only by maternal or fetal abnormalities, but also by placental factors, such as uteroplacental insufficiency. The pathophysiology of uteroplacental insufficiency is still not well understood. One of the suggested mechanism is atherosclerosis of basal arterioles and diffuse lipid infiltration of the placental bed, leading to an obstructive vasculopathy^6^. This abnormal accumulation of lipid could result in pathological placental inflammation and dysfunction, which in turn reduces the transportation of nutrients, including lipids and amino acids. This could lead to inadequate weight gain of the fetus, which may result in SGA^7^.

Dyslipidemia, defined as high levels of low-density lipoprotein cholesterol (LDL-C) and triglyceride (TG) or a low levels of high-density lipoprotein cholesterol (HDL-C), is known to act a central role in the pathogenesis of atherosclerosis and adult cardiovascular disease^8,9^. Moreover, modifying dyslipidemia has protective effects against further disease progression^10,11^. During pregnancy lipid parameters change due to adaptation to physiological metabolism^12^. Even though the first trimester of pregnancy is a very important period for placental implantation and formation, pathologic change of lipid parameter and its biosynthesis is still not well known. There are only a few preceding studies on this topic without consistent findings; one study reported there was no association between SGA and the lipid level during the first trimester^13^ whereas another study reported high HDL-C level in early pregnancy was associated with lower birth weight^14^. Overall, the results on the effect of dyslipidemia during pregnancy on fetal growth has been conflicting^7,13–15^.

As quantitative profiling of cholesterol and its precursors/metabolites can provide insights into metabolic signatures of biologically active cholesterol^16,17^, we aimed to evaluate the relationship between maternal cholesterol levels in lipid profiles and metabolic profiling in the first trimester and subsequent risk for small-for-gestational-age birthweight (SGA).

## Results

Among pregnant women who were enrolled during the study period, 1337 women met the inclusion criteria and were included in the analysis. In the study population, 107 neonates (8.0%) were delivered with birthweight less than 10 percentile and were classified as SGA group. Table 1 shows the maternal characteristics of the study population. Maternal characteristics including age and parity were not significantly different between SGA and non-SGA group. However, maternal height, weight/BMI at the time of 10–14th weeks of blood sampling were significantly lower in the SGA group than the non-SGA group (*p* < 0.05). In terms of pregnancy outcomes, women who delivered SGA neonates had higher frequency of pregnancy associated hypertension (gestational hypertension or preeclampsia) on trend but did not reach statistical significance. And the risk of gestational diabetes was not different between the two groups of cases.

**Table 1:** Maternal characteristics and pregnancy outcomes of study population.

	Non-SGA (n = 1230)	SGA (n = 107)	*p*
**Maternal characteristics**			
Age, years*	32 (30–35)	32 (29–35)	0.929
Primiparous, n (%)	53 (646/1219)	51 (54/107)	0.616
Height, cm*	161 (158–165)	160 (157–163)	0.001
Weight at blood sampling, kg*	57 (52–64)	54 (50–60)	0.000
BMI at blood sampling*	22 (20–24)	21 (19–23)	0.004
**Pregnancy outcomes**			
GDM, %(n)	6.3 (77)	4.7 (5)	0.675
pregnancy associated hypertension^§^, %(n)	1.2 (15)	3.7 (4)	0.059
Gestational age at delivery, weeks*	39.1 (38.3–40.0)	39.3 (38.3–40.1)	0.518
Birth weight*	3.3 (3.1–3.5)	27 (2.6–2.8)	0.000

BMI; body mass index, GDM; Gestational diabetes mellitus, SGA; small for gestational age.

*All values were presented by median (interquartile ranges).

^§^ Includes gestational hypertension and preeclampsia.

**Figure 1:**
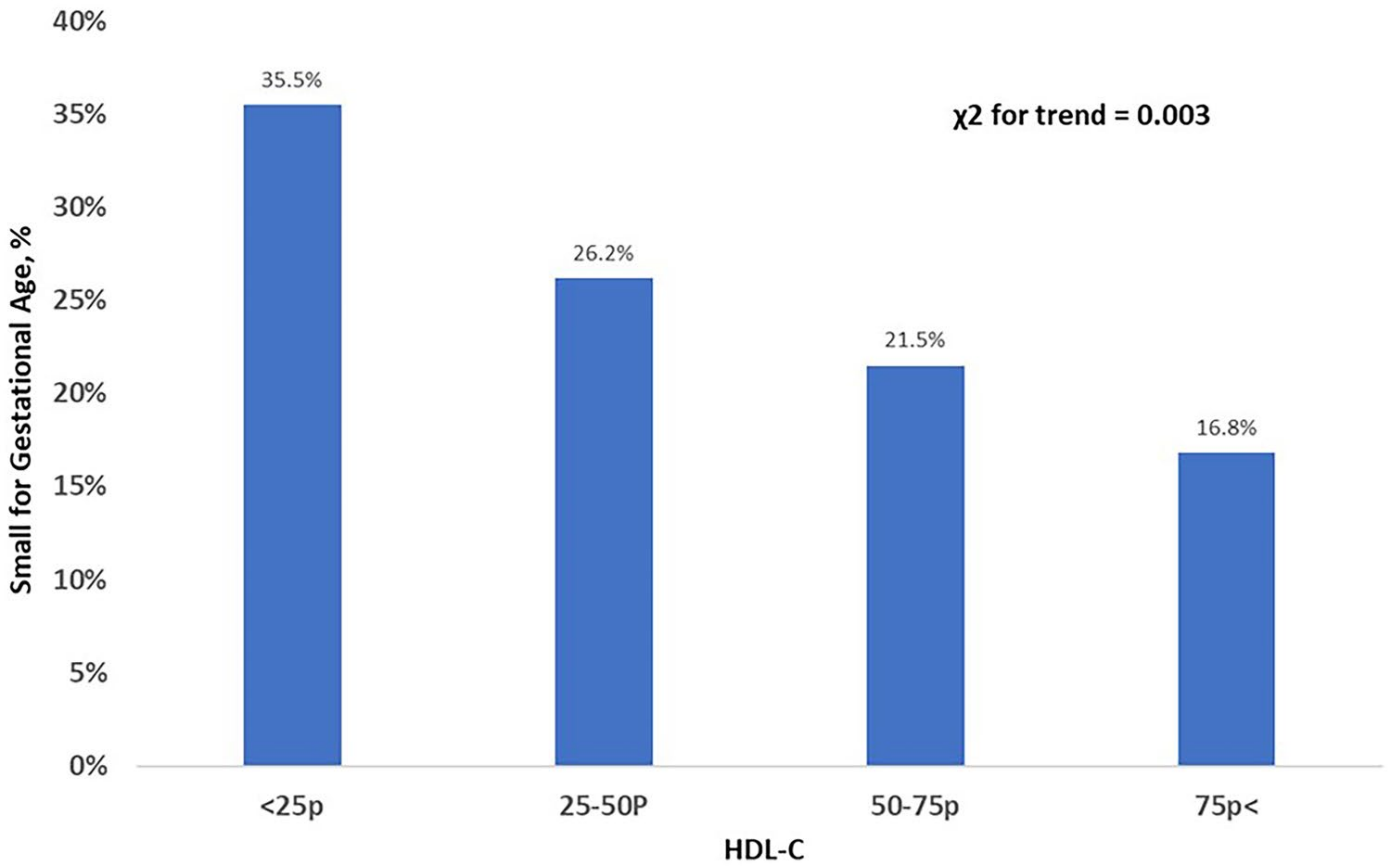
The risk for SGA according to maternal serum high-density lipoprotein cholesterol (HDL-C) quartiles at 10–14 weeks of gestational age.

Table 2 compares lipid parameters in maternal serum at 10–14 weeks between the two groups. Maternal HDL-C levels at first trimester were significantly lower in women who delivered SGA neonates than those who did not (*p* = 0.022), but other lipid profiles were not significantly different. As shown in Fig. 1, the SGA showed negative correlation with maternal serum HDL-C quartiles [HDL-C < 25th percentile 35.5%, 25th ≤ HDL-C < 50th percentile 26.2%, 50th ≤ HDL-C < 75th percentile 21.5%, HDL-C ≥ 75th percentile 16.8%, chi-square for trend, *p* = 0.003).

**Table 2:** Comparison of lipid parameters and glucose in maternal serum at gestational age of 10–14 weeks under fasting condition.

	non-SGA (n = 1230)	SGA (n = 107)	*p*
Total cholesterol, mg/dL	173 (154–192)	166 (151–190)	0.129
TG, mg/dL	104 (82–136)	100 (82–126)	0.098
HDL-C, mg/dL	69 (59–78)	65 (55–74)	0.022
HDL-C < 25 percentile, % (n)	24 (293/1230)	36 (38/107)	0.007
LDL-C, mg/dL	82 (66–96)	80 (67–99)	0.978
Fasting glucose, mg/dL	80 (74–86)	81 (73–86)	0.796
Adiponectin, ng/mL*	5036 (2962–7762)	6445 (3695–10,609)	0.075
Free fatty acid, uEq/L^§^	583 (444–742)	557 (375–708)	0.335

TG, triglyceride; HDL-C, high-density lipoprotein cholesterol; LDL-C, low-density lipoprotein cholesterol.

All values were presented by median (interquartile ranges).

* Available in 625 women (577 in non-SGA group, 48 in SGA group).

^§^ Measured in 476 women (436 in non-SGA group, 40 in SGA group).

Table 3 shows the relationship between low HDL-C levels and the risk of SGA after adjustment. Low HDL-C levels (< 25 percentile) were significantly associated with the risk of SGA, adjustment for maternal age, height, and weight at blood sampling (model 1). This relationship remained significant even after adjustment for the development of pregnancy associated hypertension (model 2). Among the study population, 625 women were measured for adiponectin in the stored blood sample taken at 10–14 weeks of gestation. The low HDL-C levels were a persistent risk factor for SGA with an additional adjustment for adiponectin (model 3).

**Table 3:** The relationship between maternal serum high-density lipoprotein cholesterol (HDL-C) at 10–14 weeks of gestational age (fasting condition) and SGA, analyzed by multiple logistic regression analysis*.

	AOR	95% CI	*P*-value
**Model 1**			
HDL-C < 25 percentile	1.849	1.1212–2.822	0.004
Maternal height	0.957	0.917–0.999	0.044
Maternal weight at blood sampling	0.959	0.934–0.984	0.001
**Model 2**			
HDL-C < 25 percentile	1.801	1.178–2.755	0.007
Maternal height	0.957	0.917–0.999	0.046
Maternal weight at blood sampling	0.956	0.931–0.981	0.001
Pregnancy associated hypertension^§^	4.432	1.342–14.636	0.015
**Model 3**			
HDL-C < 25 percentile	2.261	1.218–4.200	0.010
Maternal height	0.952	0.893–1.016	0.139
Maternal weight at blood sampling	0.952	0.912–0.994	0.024
Pregnancy associated hypertension^§^	9.575	1.651–55.252	0.012
Adiponectin	1.073	1.006–1.144	0.032

* Via enter method after adjustment for clinical and other metabolic serum markers including maternal height and weight at blood sampling, presence of gestational hypertension, and adiponectin.

^§^ Includes gestational hypertension or preeclampsia.

Comparative metabolic signatures of cholesterols between SGA and non-SGA groups showed the significantly increased serum cholesterol/lathosterol ratio in SGA group rather than non-SGA group (*p* < 0.05, Table 4), suggesting increased endogenous cholesterol biosynthesis.

**Table 4:** Comparison of quantitative results of serum metabolic signatures of cholesterol between non-SGA and SGA groups.

	Non-SGA (n = 56)	SGA (n = 56)	*p*-value*
Cholesterol, μg/mL	683.5 (545.2–807.9)	651.1 (554.0–806.6)	0.787
Sitosterol, μg/mL	0.8 (0.6–1.2)	0.9 (0.6–1.2)	0.753
Campesterol, μg/mL	0.9 (0.7–1.4)	0.9 (0.7–1.3)	0.507
Stigmasterol, μg/mL	0.2 (0.1–0.2)	0.2 (0.1–0.2)	0.458
Desmosterol, ng/mL	48.9 (40.6–54.6)	48.9 (41.8–58.4)	0.422
7-Dehydrocholesterol, ng/mL	66.3 (55.4–78.2)	65.7 (57.2–85.0)	0.569
Lathosterol, ng/mL	339.9 (205.0–498.6)	271.8 (181.7–394.0)	0.345
Lanosterol, ng/mL	65.2 (46.7–89.2)	60.4 (40.1–81.3)	0.399
7α-OHC, ng/mL	23.3 (18.9–32.0)	23.9 (15.8–33.8)	0.887
7β-OHC, ng/mL	14.6 (13.4–16.2)	14.2 (13.2–16.2)	0.845
7-Ketocholesterol, ng/mL	18.0 (14.2–27.5)	14.9 (12.8–23.0)	0.157
27-OHC, ng/mL	11.4 (9.0–15.1)	12.3 (10.0–16.3)	0.23
24-OHC, ng/mL	18.0 (15.4–22.8)	18.2 (14.1–23.3)	0.766
4β-OHC, ng/mL	12.5 (10.1–14.6)	12.1 (9.0–14.7)	0.953
Cholesterol/DES	14.2 (12.7–16.4)	13.4 (11.8–16.2)	0.264
Cholesterol/7-DHC	10.2 (9.2–11.5)	10.1 (8.7–11.6)	0.533
**Cholesterol/lathosterol**	2.1 (1.5–2.9)	2.7 (1.6–3.7)	**0.034**
Cholesterol/lanosterol	10.9 (8.4–14.1)	10.8 (8.8–15.6)	0.103
Sitosterol/cholesterol	1.24 (1.0–1.7)	1.4 (1.0–1.8)	0.839
Campesterol/cholesterol	1.4 (1.0–1.8)	1.5 (1.0–1.9)	0.535
Stigmasterol/cholesterol	0.2 (0.2–0.3)	0.3 (0.2–0.3)	0.268
Stigmasterol/lathosterol	0.5 (0.3–0.8)	0.7 (0.4–0.9)	0.102
7α-OHC/cholesterol	3.5 (2.7–5.0)	2.9 (2.2–5.5)	0.572
7β-OHC/cholesterol	2.2 (1.8–2.8)	2.2 (1.7–2.8)	0.896
7-KC/cholesterol	3.1 (1.7–3.9)	2.3 (1.8–3.4)	0.268
27-OHC/cholesterol	1.8 (1.4–2.0)	1.9 (1.5–2.3)	0.118
24-OHC/cholesterol	2.7 (2.2–3.3)	2.6 (2.2–3.1)	0.4
4β-OHC/cholesterol	1.7 (1.2–2.1)	1.7 (1.4–2.0)	0.764
7α-OHC/7β-OHC	164.0 (127.3–202.2)	167.2 (114.5–205.4)	0.873

All values were presented by median (interquartile ranges).

*Independent *t*-tests and Mann–Whitney U test where appropriate.

## Discussion

The principal findings of this study were: (1) the levels of maternal HDL-C at 10–14 weeks in SGA neonates were significantly lower than those of non-SGA-group; (2) the risk for SGA showed negative relationship with maternal serum HDL-C quartiles at 10–14 weeks of gestational age; (3) for metabolic signature of maternal cholesterol at 10–14 weeks, SGA group higher ratio of cholesterol/lathosterol, suggesting increased endogenous cholesterol biosynthesis.

Cholesterol is an essential component of cell membranes and plays biochemical roles as metabolic precursors of steroid hormones, transmembrane signaling and cell proliferation. However, abnormally alternated cholesterol mechanisms could lead to atherosclerotic diseases such as cardiovascular and cerebrovascular diseases.

It is well known that the level of cholesterol is increased during pregnancy; however, the role of cholesterol for fetal birth weight is not well established. Although several previous studies have investigated the association between birth weight and maternal dyslipidemia in late pregnancy^18–21^, only a few studies investigated the association between maternal cholesterol early in pregnancy^15^. Nonetheless, these studies did not provide consistent results^7^. However, there are various definitions on SGA, FGR, low birth weight in neonates, and the association between maternal dyslipidemia and birthweight needs to be interpreted with caution.

Early pregnancy is a critical period for placental formation^13,15,21^, and changes in the placenta in response to the maternal environment in early pregnancy could lead to substantial structural and functional alterations. And changes of placental function could also contribute to the birth weight of fetus. In the current study, we showed that maternal dyslipidemia and altered endogenous cholesterol biosynthesis in early pregnancy were associated with subsequent risk of SGA.

In SGA group, maternal HDL-C was decreased at the first trimester, and cholesterol/lathosterol ratio was significantly increased. Lathosterol is one of upstream precursors of cholesterol and its concentration has been represented in the whole-body cholesterol biosynthesis^22,23^. The result of the current study in early pregnancy is consistent with the previously findings on the association between cholesterol in late pregnancy and fetal growth^24^.

The authors attempted to find out the association between dyslipidemia and altered cholesterol metabolism and SGA. Considering the fundamental effect of dyslipidemia in adult atherosclerosis, it may be possible that altered cholesterol biosynthesis and dyslipidemia with decreased HDL-C might result in atherosclerotic placental change. Atherosclerotic placental change could lead to reduction in material blood flow to the fetus, thereby decreasing nutrient supply to the fetus and interfering fetal growth^25^.

As the current study showed the impact of dyslipidemia in early pregnancy, it has several clinical implications. First, low level of maternal HDL-C in early pregnancy may be used as a predictive marker for fetal growth restriction. In Table 3, women with low HDL-C (< 25 percentile) had increased risk for SGA with adjusted odds ratio of 1.801–2.261. However, evaluating the performance of dyslipidemic markers on fetal growth was needed, which is beyond of the scope of the current study.

Next, noting the effects of dyslipidemia in early pregnancy on fetal growth also raises interests on the possible preventive target for SGA. In adults, cholesterol lowering agents have been shown to decrease the risk of subsequent atherosclerosis^26^. As previous studies failed to show preventive effect of aspirin against fetal growth restriction in spite of its protective effect on preeclampsia^27,28^, lipid modulating medications may be another candidate for prevention of SGA. Indeed, cholesterol lowering agent such as—pravastatin are currently being investigated as target agents for prevention of preeclampsia^29,30^, which is another component of uteroplacental insufficiency.

The current study evaluated the relationship between maternal cholesterol levels and its biologically active precursors and metabolites in early pregnancy and the subsequent risk for SGA. Further studies are needed to include a wider spectrum of study population ranging from preconception to delivery and to investigate the birth weights according to altered cholesterol mechanism. The current study found the risk of SGA from dysplipidemia in the first trimester; further studies are also needed to investigate the effect of cholesterol modulating agent in preventing SGA.

SGA was defined as birth weight of less than 10 percentile. Although this definition is not exactly in line with the definition of FGR, the former means constitutionally small, and the latter means pathologically small. Therefore, the limitations of current study are (1) SGA was defined as birth weights of less than 10 percentile; (2) the lack of information on the number of FGR and SGA cases in the group of newborns below 10 percentiles; and (3) both large and appropriate for gestation age groups were included as the control group. To our knowledge, this is the first study to examine the association between dyslipidemia and cholesterol biosynthesis in early pregnancy and SGA. This study was conducted from a prospective cohort study which collected fasting maternal blood in early pregnancy and followed up till delivery. However, this study included only Korean pregnant women, more studies in other ethnicities/races are needed to confirm of the current study. In addition, further studies for association between SGA and periconceptional HDL-C may also help the understanding the relationship between dyslipidemia and fetal health.

In conclusion, maternal dyslipidemia and increased cholesterol biosynthesis caused delivery of SGA neonates even in early pregnancy.

## Materials and methods

### Study design

This is a secondary analysis of a prospective cohort study of Korean pregnant women which was designed to examine the clinical significance of nonalcoholic fatty liver disease (NAFLD) during pregnancy. Among the women enrolled in this cohort, the women who met the following criteria were included in the current study: (1) collection of maternal serum in the first trimester after fasting at least 8 h; (2) followed up till delivery; (3) delivered in term gestation (≥ 37 weeks of gestation) from May 2015 to May 2020. The pregnant woman with pre-existing diabetes or chronic hypertension and the neonate who had major malformations were excluded.

In this cohort, maternal fasting blood was routinely taken at 10–14 weeks’ gestation and lipid parameters were measured. The lipid parameters were compared between women who delivered SGA neonates (SGA group) and women who did not (non-SGA group). In addition, metabolic signatures of cholesterol in serum were evaluated in a subset of 56 women in SGA group and 56 women in non-SGA group. The Institutional Review Board of in Seoul Metropolitan Government Seoul National University Boramae Medical Center (IRB No. 1308–116-518), Seoul National University College of Medicine/Seoul National University Hospital (IRB No. 2010-053-1163), and Public Institutional Review Board Designated by Ministry of Health and Welfare (P01-201404-BM-03) approved the study and all patients provided written informed consent. All methods were performed in accordance with the ethical standards of our institutional research committee and with the 1964 Helskinki declaration and its later amendments.

### Definition of SGA

All neonates were measured for birthweight at the time of delivery. SGA was defined as birthweight < 10th percentile for gestational age, according to the Korean reference^31^.

### Lipid parameters measurement

Maternal fasting blood was taken at 10–14 weeks’ gestation and obtained blood was transferred into serum-separating tube. Serum was measured for lipid parameters including triglyceride, total cholesterol (TC), high-density lipoprotein, and low-density lipoprotein, by a clinical chemistry analyzer (Roche/Hitachi, Japan). After centrifugation, serum samples were stored at − 70 °C for further analysis.

### Measurement of precursors and metabolites of serum cholesterol

To compare the metabolic signatures of maternal serum cholesterol in the first trimester between SGA and non-SGA group, a subset was selected consisting of 56 women from SGA group and 56 control women from on-SGA group selected after propensity score matching. The maternal age, parity, height, weight at sampling (10–14 weeks), gestational age at sampling, and presence or absence of hypertension during pregnancy were adjusted for propensity score matching.

### Metabolic signatures of cholesterols

Sample preparation was performed with previous methods^17,32^. Briefly, 20 μL serum samples spiked with 20 μL of an IS mixture (*d*_6_-cholesterol, 100 μg/mL; *d*_7_-7β-OH-cholesterol, 2 μg/mL; *d*_6_-27-OH-cholesterol, 4 μg/mL) were added to 0.5 mL methanol and shaked using a TissueLyser (Qiagen; Hilden, Germany) at 25 Hz for 1 min. After centrifugation at 12,000 rpm for 10 min, the supernatant was loaded onto the H-PPT cartridges and eluted with 0.5 mL MeOH three times. The eluate was evaporated under N_2_ evaporator at 40 °C and dried in a vacuum desiccator at least 30 min. The dried extracts were derivatized with 40 μL *N*-methyl-*N*-(trimethylsilyl)trifluoroacetamide /ammonium iodide/dithioerythritol (500:4:2, *v/w/w*) at 60 °C for 20 min and the aliquot (2 μL) was injected into gas chromatography–mass spectrometric (GC–MS) system.

GC–MS was performed using an Agilent 7890A Plus gas chromatograph interfaced with a single quadrupole Agilent 5975C MSD (Agilent Technologies; Palo Alto, CA, USA) at an electron energy of 70 eV and an ion source temperature of 280 °C. Each sample (2 μL) was injected in a split mode (10:1) at an injection temperature of 280 °C. Separation of sterols was achieved on an Ultra-2 capillary column (20 m × 0.2 mm I.D. × 0.33 µm film thickness; Agilent Technologies). The GC oven temperature was initially set at 160 °C. It was then ramped to 260 °C at 20 °C/min and held for 5 min, ramped to 280 °C at 2 °C /min, then finally ramped to 320 °C at 5 °C/min and held for 4 min. The carrier gas was helium at a constant flow of 1.2 mL/min.

For quantitative analysis of cholesterol and its 4 precursors and 6 oxygenated metabolites as well as 3 plant sterols, identification of GC–MS peaks was achieved by comparing retention time and matching height ratios of characteristic ions with those of corresponding standards (Supplementary Table 1). All analytes were detected in electron impact ionization with the selected-ion monitoring (SIM) mode.

### Statistical analysis

All data were analyzed using commercially available software (SPSS, version. 23, Chicago, IL, USA; R statistical packages, version 3.4.4, R Foundation, Vienna, Austria, www. R-project.org). Proportions were compared using Fisher’s exact test, and comparisons of continuous variables between groups were performed with independent t-test and Mann–Whitney U test where appropriate after testing for normal distribution of continuous data using Shapiro–Wilk test. For multivariate analysis, logistic regression analysis with enter method was conducted. In the logistic regression analysis, confounding variables were chosen from the univariate analysis; variables with *p*-values of < 0.05 in the univariate analysis were entered into stepwise selection to build a model. Quantitative results are expressed as median (interquartile range) and statistical significance was considered at *p* < 0.05.

## Supplementary Information

Supplementary Table 1.
