# Supplementary material for: Maternal dyslipidemia and altered cholesterol metabolism in early pregnancy as a risk factor for small for gestational age neonates
Source: Sci Rep. 2021 Oct 26;11:21066. doi: 10.1038/s41598-021-00270-1 (PMC8548295; doi:10.1038/s41598-021-00270-1)
Supplement: Supplementary file 1 — Supplementary Table 1. [file 41598_2021_270_MOESM1_ESM.docx]

**Maternal dyslipidemia and altered cholesterol metabolism in early pregnancy**

**as a risk factor for small for gestational age neonates**

So Yeon Kim,^1,2^ Seung Mi Lee,^1^ Go Eun Kwon,^3^ Byoung Jae Kim,^1,4^ Ja Nam Koo,^5^ Ig Hwan Oh,^5^ Sun Min Kim,^1,4^ Sue Shin,^6,7^ Won Kim,^8,9^ Sae Kyung Joo,^8,9^ Errol R. Norwitz,^10^ Young Mi Jung, ^1^ Chan-Wook Park,^1^ Jong Kwan Jun,^1^ Man Ho Choi,^3^ Joong Shin Park^1^

^1^ Department of Obstetrics and Gynecology, Seoul National University College of Medicine, Seoul, Korea

^2^ Department of Obstetrics and Gynecology, University of Ulsan College of Medicine, Asan Medical Center, Seoul, Korea

^3^ Molecular Recognition Research Center, Korea Institute of Science and Technology, Seoul, Korea

^4^ Department of Obstetrics and Gynecology, Seoul Metropolitan Government Seoul National University Boramae Medical Center, Seoul, Korea

^5^ Seoul Women's Hospital, Incheon, Korea

^6^ Department of Laboratory Medicine, Seoul National University College of Medicine, Seoul, Korea

^7^ Department of Laboratory Medicine, Seoul Metropolitan Government Seoul National University Boramae Medical Center, Seoul, Korea

^8^ Department of Internal Medicine, Seoul National University College of Medicine, Seoul, Korea

^9^ Department of Internal Medicine, Seoul Metropolitan Government Seoul National University Boramae Medical Center, Seoul, Korea

^10^ Department of Obstetrics and Gynecology, Tufts University School of Medicine, Boston, MA, U.S.A

SY Kim and SM Lee contributed equally as the first authors of this study.

JS Park and MH Choi contributed equally as co-corresponding authors.

**Correspondence to:**

**Joong Shin Park, MD, Ph.D.**

Department of Obstetrics and Gynecology,

Seoul National University College of Medicine,

101 Daehak-Ro, Jongno-Gu, Seoul 03080, Korea.

Tel: +82-2-2072-2380, Fax: +82-2-762-3599,

E-mail: [jsparkmd@snu.ac.kr](mailto:jsparkmd@snu.ac.kr)

**Man Ho Choi, Ph.D.**

Molecular Recognition Research Center,

Korea Institute of Science and Technology

5 Hwarang-ro 14-gil, Seoul 02792, Korea

Tel: +82-2-958-5081, Fax: +82-2-958-5059;

E-mail: [mh_choi@kist.re.kr](mailto:mh_choi@kist.re.kr)

**Supplementary table 1.** Maternal characteristics, pregnancy outcomes and HDL-C of study population after propensity score matching

|  | Non-SGA (n=56) | SGA (n=56) | *p* |
| --- | --- | --- | --- |
| Maternal characteristics |  |  |  |
| Age, years^*^ | 33 (30-34) | 33 (30-35) | 0.744 |
| Height, cm^*^ | 159 (157-162) | 160 (156-163) | 0.353 |
| Weight at blood sampling, kg^*^ | 54 (50-58) | 54 (49-61) | 0.968 |
| Pregnancy outcomes |  |  |  |
| GDM, %(n) | 0 (0) | 0 (0) | N/A |
| pregnancy associated hypertension, %(n)^§^ | 0 (0) | 0 (0) | N/A |
| Gestational age at delivery, weeks^*^ | 39.0 (38.2-40.2) | 39.5 (38.5-40.2) | 0.353 |
| HDL-C, mg/dL^*^ | 73 (61-82) | 65 (55-72) | 0.002 |

Abbreviations: BMI; body mass index, GDM; Gestational diabetes mellitus, SGA; small for gestational age, N/A; not applicable

^*^All values were presented by median (interquartile ranges)

^§^ Includes gestational hypertension and preeclampsia
